# No effect of artificial light of different colors on commuting Daubenton's bats (*Myotis daubentonii*) in a choice experiment

**DOI:** 10.1002/jez.2178

**Published:** 2018-05-29

**Authors:** Kamiel Spoelstra, Jip J. C. Ramakers, Natalie E. van Dis, Marcel E. Visser

**Affiliations:** ^1^ Department of Animal Ecology Netherlands Institute of Ecology (NIOO‐KNAW) Wageningen The Netherlands; ^2^ Plant Ecology and Nature Conservation Group Wageningen University Wageningen The Netherlands

**Keywords:** artificial light at night, bats, light color, light pollution, *Myotis daubentonii*

## Abstract

Progressive illumination at night poses an increasing threat to species worldwide. Light at night is particularly problematic for bats as most species are nocturnal and often cross relatively large distances when commuting between roosts and foraging grounds. Earlier studies have shown that illumination of linear structures in the landscape disturbs commuting bats, and that the response of bats to light may strongly depend on the light spectrum. Here, we studied the impact of white, green, and red light on commuting Daubenton's bats (*Myotis daubentonii*). We used a unique location where commuting bats cross a road by flying through two identical, parallel culverts underneath. We illuminated the culverts with white, red, and green light, with an intensity of 5 lux at the water surface. Bats had to choose between the two culverts, each with a different lighting condition every night. We presented all paired combinations of white, green, and red light and dark control in a factorial design. Contrary to our expectations, the number of bat passes through a culvert was unaffected by the presence of light. Furthermore, bats did not show any preference for light color. These results show that the response of commuting Daubenton's bats to different colors of light at night with a realistic intensity may be limited when passing through culverts.

## 1 INTRODUCTION

Artificial light has increased dramatically over the last decades and is now ubiquitous in virtually all populated areas worldwide (Falchi et al., 2016; Kyba et al., 2017). The increase in artificial light is widely recognized as a threat to biodiversity and ecosystems (Gaston, Bennie, Davies, & Hopkins, 2013; Hölker, Wolter, Perkin, & Tockner, 2010; Rich & Longcore, 2006). Information on the impact on different species groups is accumulating, but there is a general need for knowledge from experimental testing in natural habitat (Gaston, Visser, & Hölker, 2015; Swaddle et al., 2015). Bats are strongly affected by light at night, and the indirect attraction of bats by accumulated insects around illumination has been known for a long time (Rydell, 1992; Rydell & Racey, 1995). This effect has more recently been shown in experimental setups (Cravens, Brown, Divoll, & Boyles, 2018; Minnaar, Boyles, Minnaar, Sole, & McKechnie, 2014; Spoelstra et al., 2017; Wakefield, Stone, Jones, & Harris, 2015). However, slow‐flying bats, such as *Myotis* and *Plecotus* species, generally avoid illumination (Furlonger, Dewar, & Fenton, 1987; Rydell, 1992; Spoelstra et al., 2017). This response is generally considered as predator avoidance (Duvergé, Jones, Rydell, & Ransome, 2000; Jones & Rydell, 1994; Rydell, Entwistle, & Racey, 1996; Zeale et al., 2016).

Illumination of linear structures in the landscape can have a strong impact on *Rhinolophus* and *Myotis* species commuting between nursery roosts and foraging habitat (Stone, Jones, & Harris, 2009, 2012). At such locations, light at night may prevent access of many individuals to foraging grounds, and has potentially a strong impact at the population level. A way to alter the response of bats to light at night is to change the spectrum. For example, lights attracting fewer insects consequently attract fewer bats, which has been shown for low‐pressure sodium light sources (Eisenbeis, 2006; Rydell, 1992; Stone, Wakefield, Harris, & Jones, 2015). In a recent experimental study, free ranging, slow‐flying *Myotis*, and *Plecotus* bat species were shown to avoid white and green light, but to be equally active in red light compared to dark control (Spoelstra et al., 2017). The cause of this response may be related to a relative high sensitivity of bat eyes to the blue part of the spectrum (Müller et al., 2009), although bats may well be able to see red light (Feller et al., 2009; Wang, 2003; Zhao et al., 2009). The knowledge on how commuting bats respond to light with different spectra is however limited and the response of bats to light when commuting may well differ from the response of foraging bats. There is some information on flight behavior of non‐light‐shy bat species, which were found to fly faster in light (Polak, Korine, Yair, & Holderied, 2011) and tend to avoid crossing open areas with higher light intensities (Hale, Fairbrass, Matthews, Davies, & Sadler, 2015, but see Stone et al., 2012). Information on the response of commuting light‐shy bats to different light spectra is limited, yet knowledge on this response is highly relevant to maintain landscape connectivity for this group, which consequently may strongly benefit from mitigation measures.

Here, we used a unique location to study the impact of light with different spectra on Daubenton's bats (*Myotis daubentonii*) commuting through two identical, parallel culverts underneath two roads. This location is preeminently suitable to test for a preference in light color in a choice experiment. Each night, we illuminated the culverts with paired combinations of light spectra (white, green, red, and dark), with a realistic light level (5 lux), comparable to light levels used in our previous study (Spoelstra et al., 2017). We expected a clear effect of light spectrum on the number of bats passing through each culvert, with bats avoiding white and green light.

## 2 METHODS

### 2.1 Experimental site

We tested the response of commuting Daubenton's bats (*M. daubentonii*) to experimental light at a location where bats pass two roads and two cycle tracks by flying through two identical, parallel culverts near Elburg in the Netherlands (52°26583′ N, 5°50.506′ E). These culverts drain a small stream, are 31 m long, and have a diameter of 1.57 m. The culverts are filled with just over a meter of water, leaving a space of approximately 50 cm between the water surface and the culvert ceiling for bats to pass. Daubenton's bats are the only species observed flying through the culverts, flying back and forth between roosts on the south side, and feeding grounds on the north side (see Supporting Information Figures S1–S4).

### 2.2 Light treatment

We attached a flat wooden frame to the ceiling, 4 m into the culverts from the Southern entrances (the side bats entered when flying from the roosts to the foraging grounds). We installed white, green, and red LED light (custom made by Philips, Eindhoven, the Netherlands), with two lamps per light color (see Supporting Information Figures S4, S5, and S8). The LED lights were adjusted such that the light intensity at the water level was 5.0 ± 0.2 lux (1 SEM) at the water level. The LED lamps did not emit any sound between 0 and 120 kHz. Light conditions were kept constant within each night, and lighting conditions were changed from night to night using a factorial design. In this lighting schedule, light treatment (dark, white, green, and red) was always different between the two culverts, and none of the two tunnels had the same light color in consecutive nights. Thereby, all light color treatments and treatment combinations were equally distributed over both culverts (see Supporting Information Figure S9 for the full schedule). Lights were on from 30 min after sunset to 30 min before sunrise, and we tested between July 1, 2015 and September 15, 2015.

### 2.3 Bat activity measurement

Passing bats were recorded with two Pettersson D500× detectors (Pettersson Elektronik AG, Sweden) in each culvert, placed directly next to the lamps on the wooden frame. In order to only include bats passing right beneath the detectors, we fitted the detectors with small (4 cm Ø) plastic tubes directing microphone sensitivity downwards (see Supporting Information Figure S6 for D500× settings and setup). Bat passes were quantified as the number of 5‐s sound files with two or more pulses of a *Myotis* species. Bat calls were identified with the SonoChiro software (Biotope Research & Development, Mèze, France). Because many *Myotis* species have similar echolocation sounds properties, the program occasionally identified *Myotis* species other than *M. daubentonii*. As no other *Myotis* species were observed flying through the culverts, we included these in our analysis. In order to have an impression of the number of individual bats passing, and the dominant flight direction, we manually counted the number of bats during five different nights throughout the experiment, using a handheld bat detector (Pettersson D100× and D240×; Pettersson, Uppsala, Sweden) and an infrared sensitive camera (Sony DCR‐SR85) with a LED infrared light (IRlamp6, David Dalton, Tucson, Arizona).

### 2.4 Statistical analysis

Statistical analysis was done with R v3.3.1 (R Core Team, 2016) with a significance level of 0.05. We used generalized linear and generalized linear mixed models using the standard glm function in R and the glmer function in the lme4 package (Bates, Mächler, Bolker, & Walker, 2015). We tested the effect of the light treatment combination on the success/failure ratio (binomial errors). Successes were defined as the number of recordings made in the focal culvert, and failures were defined as the number of recordings in the alternative culvert. Light treatment combination had six levels (dark–white, dark–green, dark–red, white–green, white–red, and green–red). We added treatment orientation (a two‐level factor indicating whether these treatment combinations were oriented east–west or west–east) as fixed effect. We subsequently compared models with and without the fixed effects using the R anova.lm function, in a Chi‐Square test on the residual sum of squares. Because the illumination of the Southern entrances of the culverts may cause bats to respond differently in the evening (when flying in from the south side) and morning (when flying in from the north side), we tested first for an interaction between the effect of light treatment combination and part of the night (evening or morning, separated at one hour after astronomical midnight) on the success/failure ratio of bats flying through the corresponding light condition in either culvert, with night number as a random effect. When such an interaction was not present, we consequently tested for an effect of treatment combination on the success/failure ratio based on the total bat passes per culvert for the entire night.

## 3 RESULTS

With handheld detectors and the infrared camera, we counted between 17 and 41 (average 26) Daubenton's bats flying into the Southern (illuminated) entrances of the culverts during five evenings (until midnight) throughout the experiment (see Supporting Information Table S1). We never observed other species than *M. daubentonii* flying through the culverts or above the stream. During the first half of the night (evening), nearly all bats flew through the culverts from south to north, and the first bats never came from the north, indicating the roosts must be located on the south side and the foraging grounds on the north side of the road. Bats occasionally flew over the road above the culverts or turned around before entering. Most bats, however, flew in a straight line into the culverts, and no bats were observed to circle or forage in front of the culvert entrances.

**Figure 1:**
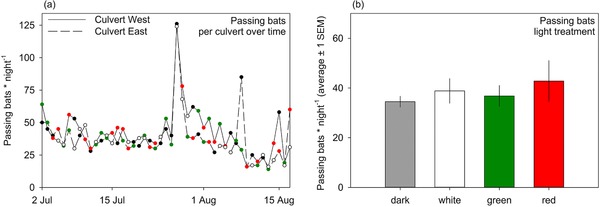
Passing bats (number of 5‐s recordings per night, including return flights). (a) Number of passing bats per night per culvert between July 2, 2015 and August 18, 2015. Colored dots indicate the nightly light color treatment of each culvert. (b) Average number of passing bats per night per treatment (raw means ± 1 SEM) [Color figure can be viewed at http://wileyonlinelibrary.com]

With the D500× detectors fitted into the culverts, we were able to continuously record passing bats for a total of 47 nights, between July 2, 2015 and August 18, 2015. Thereafter, the number of bats quickly declined. The light treatment combinations dark–white, dark–green, dark–red, white–green, white–red, and green–red were present during 8, 9, 7, 8, 8, and 7 nights, respectively. Due to a broken contact, one of the two detectors in the eastern culvert malfunctioned. The recordings of the two detectors in the western culvert were however nearly identical, so we only included the recordings of the detectors placed directly near the lights on the entrance side (see also Supporting Information Figures S4 and S5). Over the entire period, we made 41.2 ± 2.3 (1 SEM) and 38.3 ± 2.7 recordings of passing bats per night in the western and eastern culvert, respectively (Figure 1).

There was no interaction between the number of bat passes in the first and second part of the night and treatment combination (*P *= 0.25), so we tested for the effect of treatment combination on the success/failure ratio of passing bats during the full night, which was also not significant (*P *= 0.22, Table 1, Figure 1). Thus, the ratio of the number of passes through the culverts was not affected by any light color combination, indicating that the different light treatments did not disturb commuting bats.

**Table 1:** Output of statistical analysis. (a) Interaction between night part (the time of night; evening/morning, split at one hour after astronomical midnight) and light treatment combination (six principal combinations: dark–white, dark–green, dark–red, white–green, white–red, and green–red) on the ratio (success/failure) of bats passing through the corresponding light treatment in either culvert, with treatment orientation (with two levels indicating whether the principal treatment combinations were oriented east–west or west–east), and with night number as a random term. (b) Effect of light treatment combination on the ratio (success/failure) of the total number of bats per night passing through the corresponding light treatment in either culvert

	df	AIC	df	Deviance	*p*
a. Effect of the interaction between night part (evening/morning) and treatment combination					
Success/failure ∼ treatment combination + night part + treatment orientation + 1|night nr	86	565.3			
Success/failure ∼ treatment combination × night part + treatment orientation + 1|night nr	81	568.6	5	–6.63	0.25
b. Effect of treatment combination					
Success/failure ∼ treatment orientation	45	321.7			
Success/failure ∼ treatment combination + treatment orientation	40	324.7	5	–6.98	0.22

AIC, akaike information criterion.

## 4 DISCUSSION

The results show that the commuting Daubenton's bats when flying through the two culverts did not respond strongly to the four different light treatment combinations. This result is remarkable and contrasts with the clear response of free‐ranging slow‐flying species to different spectra of streetlights with comparable light levels in foraging habitat (Spoelstra et al., 2017). It also contrasts with the strong response commuting *Myotis* species showed when exposed to light along hedgerows (Stone et al., 2012). However, the number of passing individuals of another species flying along aquatic infrastructure, the pond bat (*Myotis dasycneme*), did not decline as a result of the exposure to light at night as well (Kuijper et al., 2008). Nevertheless, Kuijper et al. (2008) did observe that the presence of light affected the flight path and the feeding activity of these bats.

The absence of a response to artificial light at night observed in our study may have several reasons. A likely explanation is that the light level we applied inside the culverts was not high enough. However, Daubenton's bats fly between 15 and 25 cm above the water surface, and hence the actual light exposure has likely been well above the 5 lux present at the water level. The light levels we used are comparable or above the light levels used by Stone et al. (2012). Possibly, bats are less sensitive to disturbance inside the culverts and continue to use these irrespective of the presence of light. We incidentally observed bats passing over the roads above, but this behavior seemingly did not change after presenting light in the culverts. Traffic noise may deter commuting bats (Bennett & Zurcher, 2013) and may have caused the bats to continue to use the culverts and to be less critical of the conditions inside.

Another reason for the lack of a response may be the way the lights were installed. We fitted these 4 m deep into the culverts from the south side and ∼25 m deep from the north side. The absence of light directly at the entrance of the culverts may have weakened the strength of the treatment, as the choice for the east or west culvert is made before bats enter. However, if the location of the light setup in the culverts is determinant for the response, an interaction between time of the night (evening or morning) and light treatment combination may be expected. This is because the flight direction is northward biased in the evening (i.e., bats flying form roosts to foraging grounds, Supporting Information Table S1), and therefore southward biased in the morning (when bats return). We did however not find such an interaction, and this hypothesis may not apply as the tunnels are perfectly straight and the lighting was clearly visible from outside the tunnels (see Supporting Information Figure S7). Lighting of the roads above the culverts may furthermore have attenuated the treatment effect. However, the culvert entrance areas were largely shielded from these lights and the light intensity directly outside the culvert entrance was less than 0.2 lux. Lastly, the response observed may be species or location specific. Daubenton's bats as a species were not separately studied by Stone et al. (2012) or Spoelstra et al. (2017) and may furthermore respond differently when flying in a narrow space, a response that has been suggested for bats flying in illuminated areas with sufficient tree cover (Mathews et al., 2015). For example, bats may not fear predation in such a situation.

We are well aware that we do not know the response of individual bats, and hence have observed the response of a single group of bats. The results may therefore only be related to this location specifically; in order to know whether this response is ubiquitous, it is essential to repeat this experiment at multiple locations. The absence of a strong response to the presence of light, and to the three light spectra, differs from the response of free‐ranging *Myotis* species in foraging habitat. It is therefore important to be cautious when extrapolating the impact of light to different circumstances, that is, different types of behavior or different types of corridors. Further research is needed for effective, situation‐specific mitigation measures.

## Supporting information

Supporting information
