# Supplementary material for: No effect of artificial light of different colors on commuting Daubenton's bats (Myotis daubentonii) in a choice experiment
Source: J Exp Zool A Ecol Integr Physiol. 2018 May 29;329(8-9):506–10. doi: 10.1002/jez.2178 (PMC6220854; doi:10.1002/jez.2178)
Supplement: Supplementary file 1 — Supporting information [file JEZ-329-506-s001.docx]

**Supplementary Information to the manuscript:**

No effect of artificial light and light colour on commuting Daubenton’s bats (Myotis daubentonii) in a choice experiment

By Kamiel Spoelstra, Jip J. C. Ramakers, Natalie E. van Dis & Marcel E. Visser

**1. Experimental location and setup**

SI Fig. 1. Experimental location. Culverts are indicated with red dotted lines; position of lighting equipment is indicated with white squares (insert). Areal Image from Google Earth, 2017

SI Fig. 2. Map of the larger area around the experimental location. 1) location of the culverts; 2) potential roost site with many suitable old beech trees; and 3) locations where bats have been observed foraging.


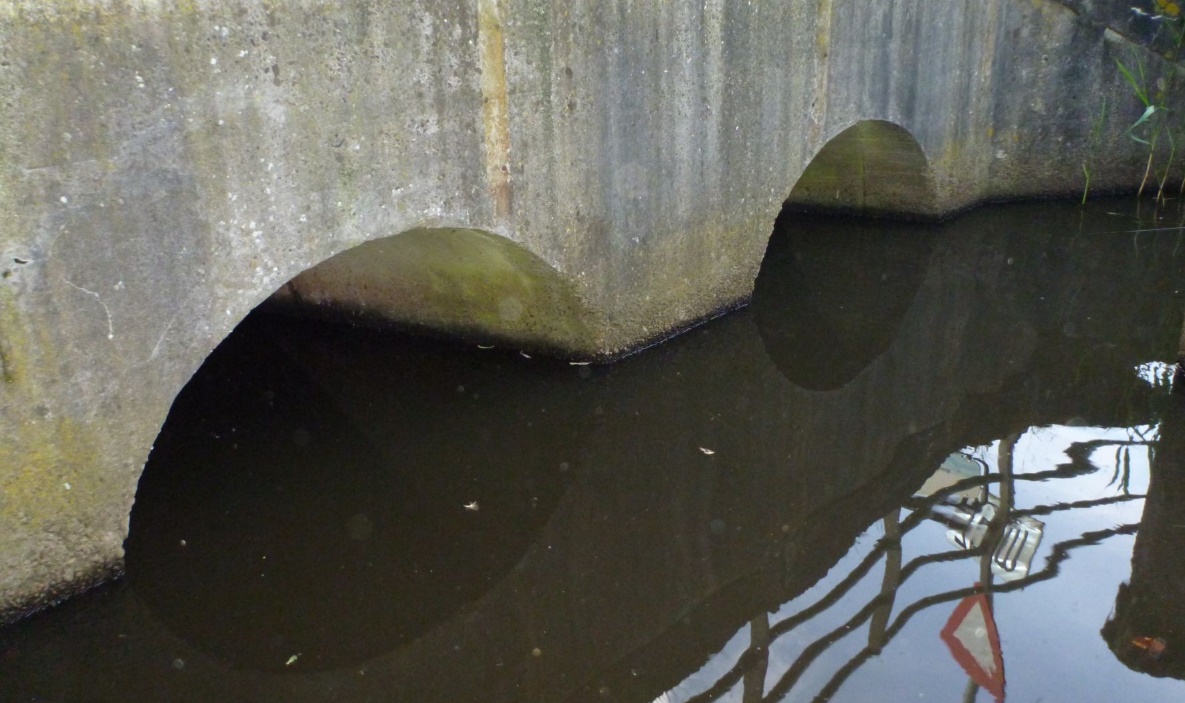


SI Fig. 3. Southern entrances of both culverts. The water level was constant throughout the experiment.

SI Fig. 4. (A) Cross-section of a culvert. (B) A plan of the wooden frame that was attached to the ceiling of each culvert (see also SI figure 4).


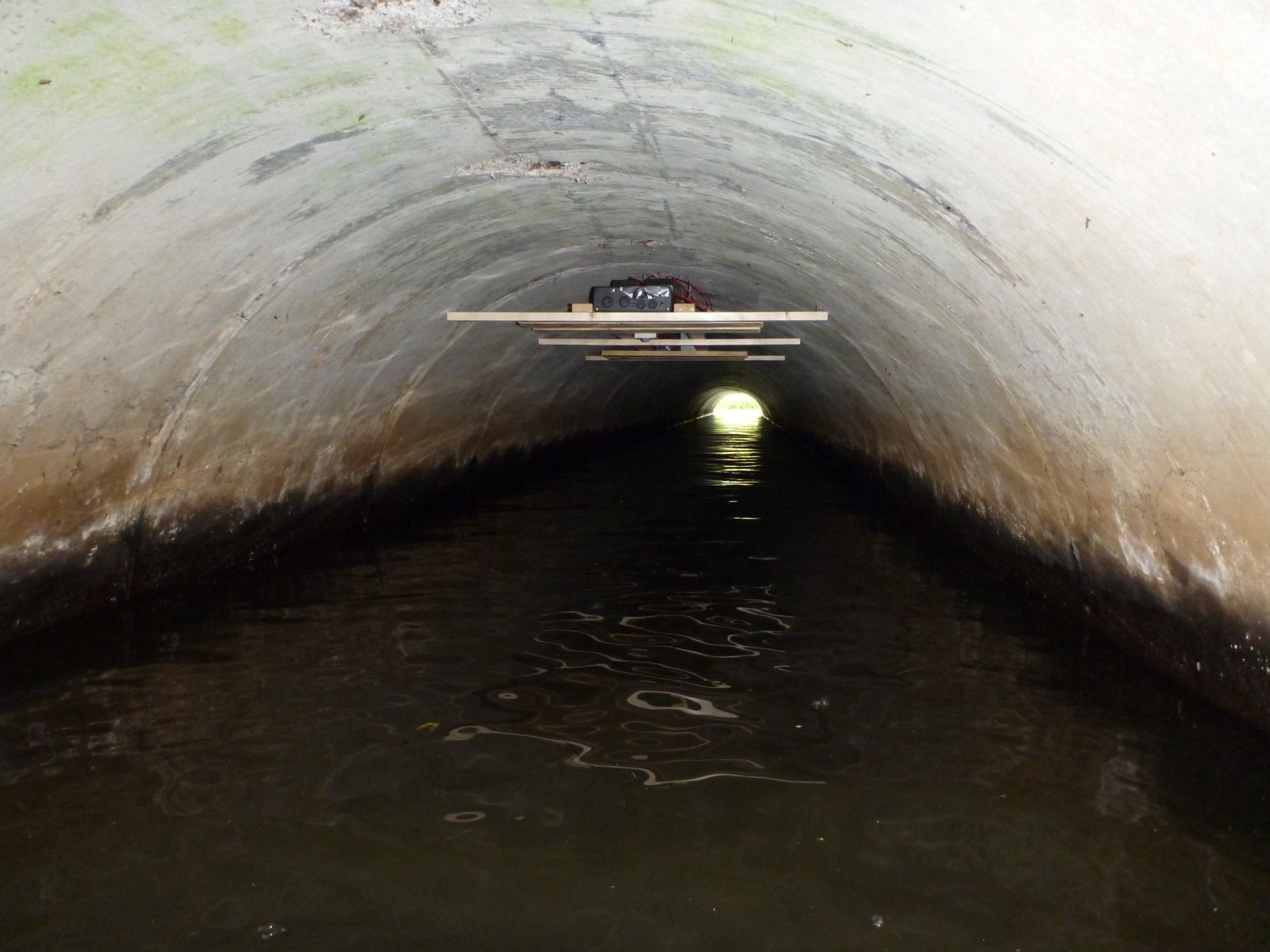


SI Fig. 5. One of the culverts with the wooden frame carrying the two detectors and the six lamps (two of each colour).


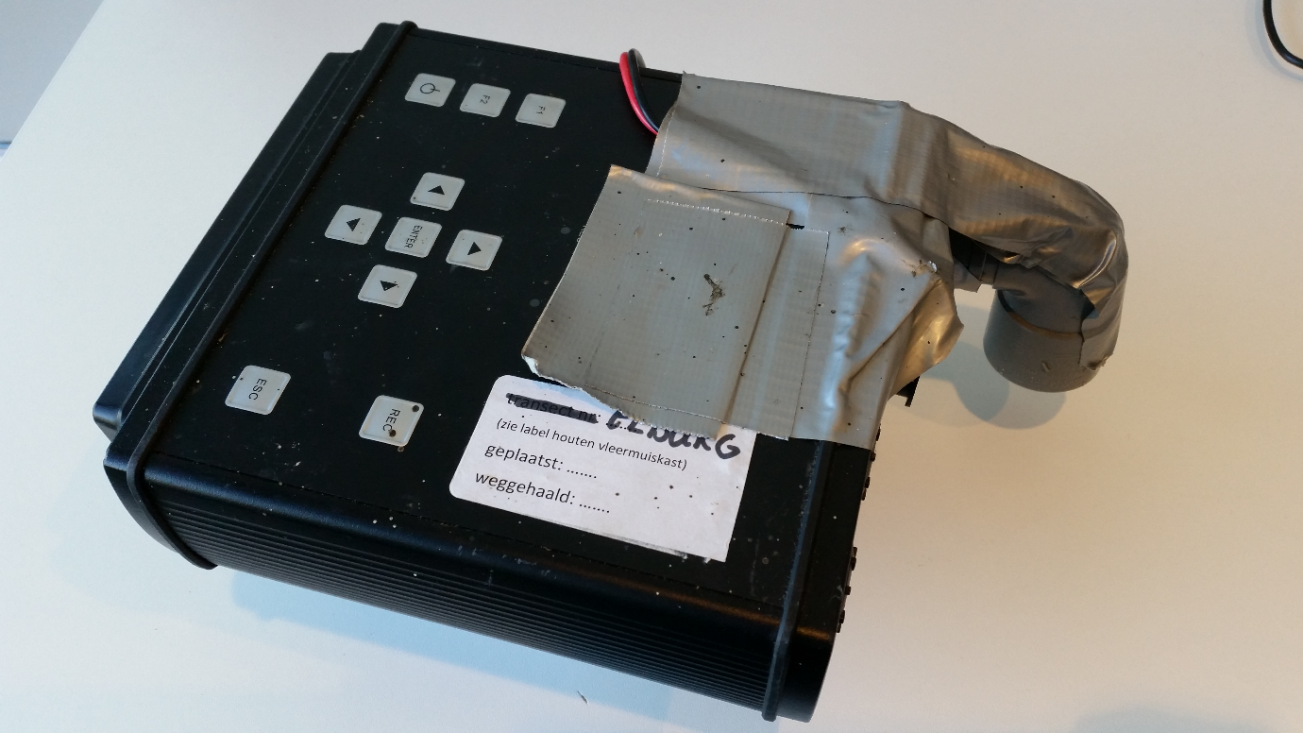


SI Fig. 6. We fitted the D500x detectors with a small 4 cm Ø pvc tube to redirect the microphone direction to only record bats flying right underneath the detectors. The detector in the setup above (SI Figure 5) is not yet equipped with such a tube. Detector displays were also shielded with tape to prevent indirect lighting (especially relevant for the dark condition).

^
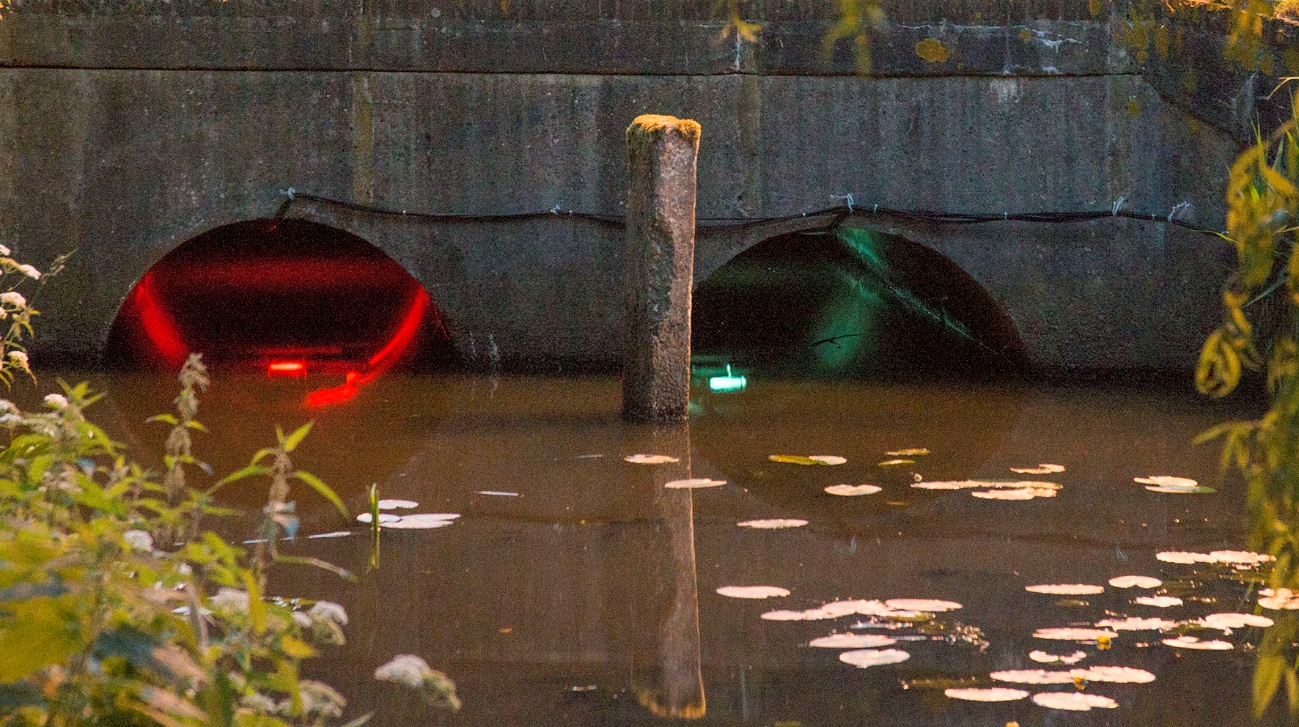
^

^
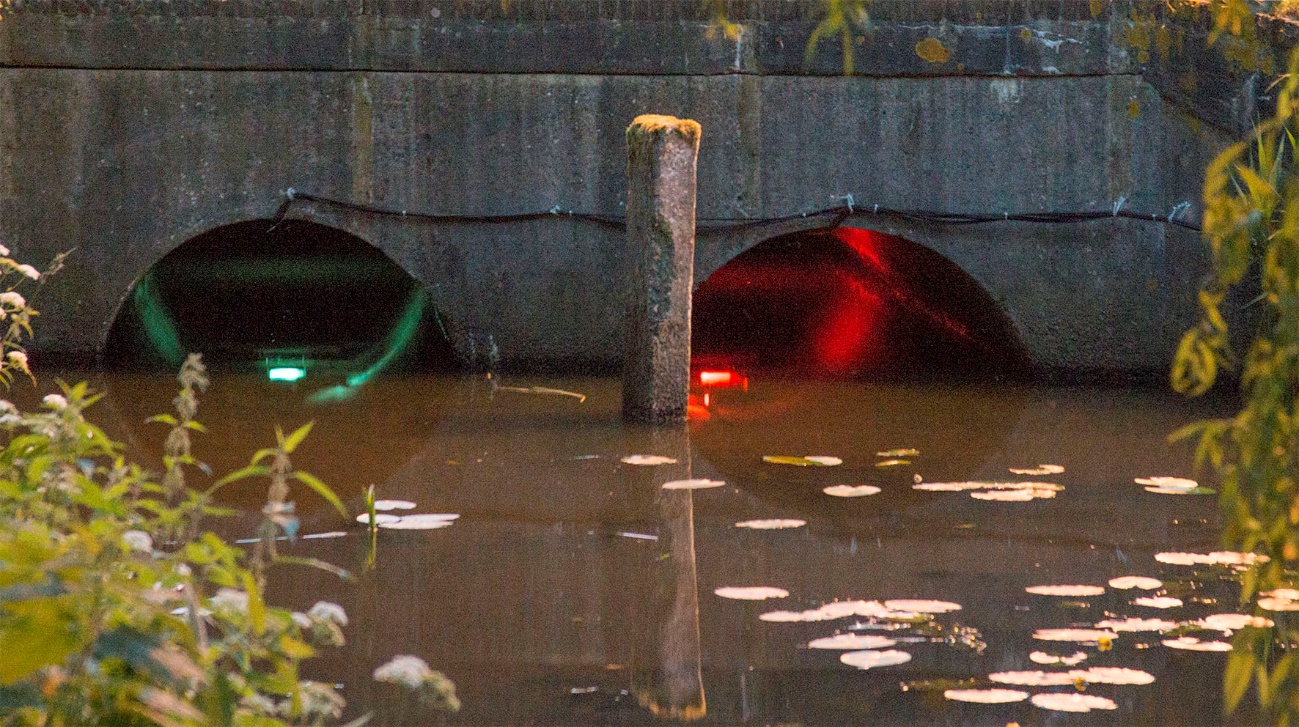
^

SI Fig. 7. Red and green illumination in the culverts. The light treatment was controlled remotely via the GSM network.

**
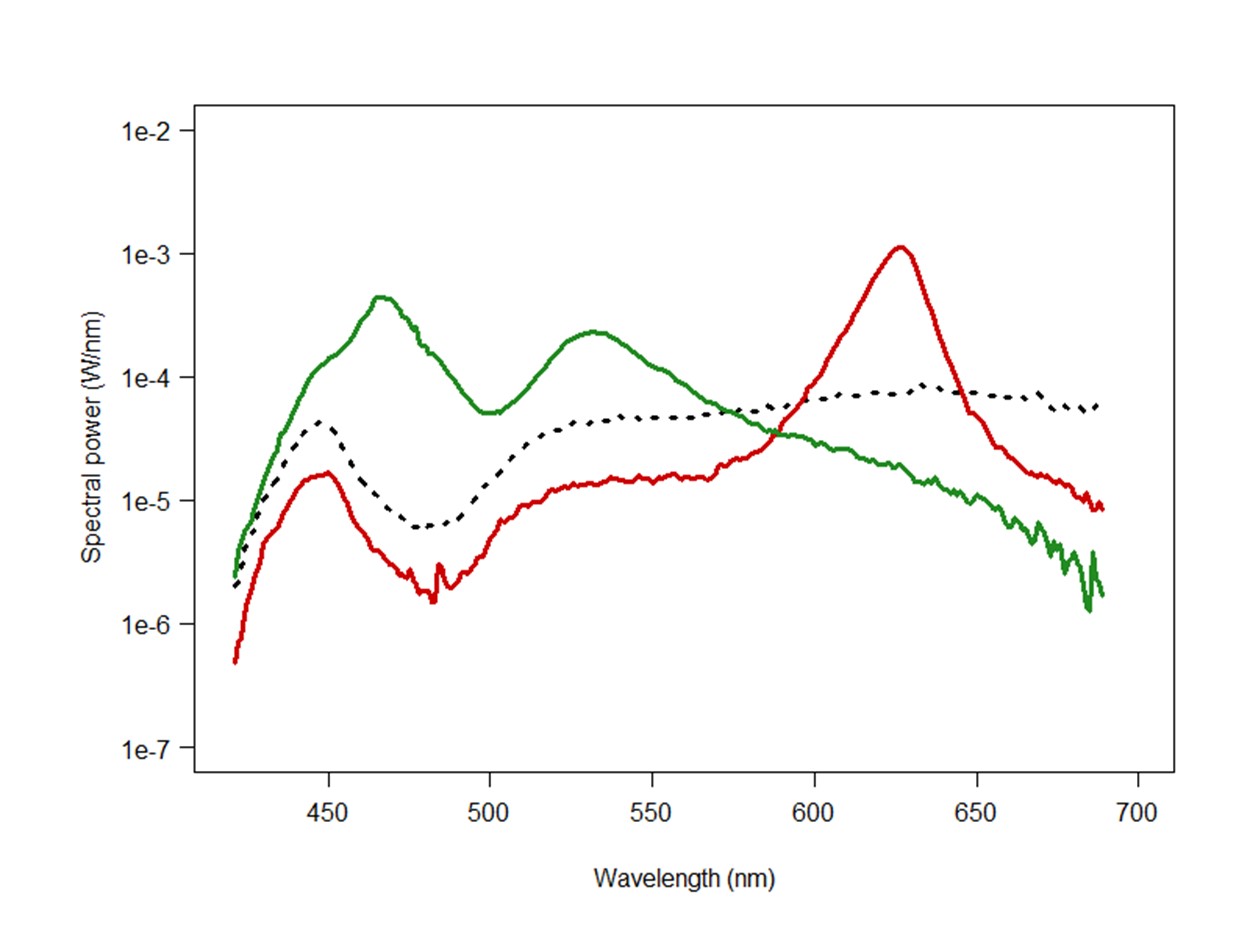
**

SI Fig. 8. Spectral composition of lamps**.** Spectral composition of the three light colours used in experiment 1. The green line corresponds to the green LED light, the red line to the red LED light, and the dashed line to the white LED light.

SI Fig. 9. Experimental lighting protocol, with per night the light colours tested for the eastern and western culvert (date indicates day before actual night).

**2. Bat Recording**

*Recording equipment*

We used Pettersson D500x detectors (Pettersson Elektronik AG, Uppsala, Sweden) with firmware version 2.D.6. The detectors were externally powered and fitted with two 32GB Compact Flash data cards (Transcend). Detectors were programmed to Profile 3 (sampling frequency = 500 kHz; pre‐trigger = off; recording length = 5 seconds; high‐pass filter = enabled (i.e. cuts off sound below 15 kHz); autorecording = enabled; trigger sensitivity = 1) and had the following settings: gain 40, trigger level 40, interval 0. With these settings, the detectors only recorded sounds above 15 kHz with an amplitude above 40 dB after being amplified with a factor of 40 (gain).

*Sound analysis*

Wave files were analysed with the SonoChiro software package (Biotope Research & Development, Mèze, France). The Northern Temperate region was selected to exclude most species not present at the experimental sites. We used the default program settings, e.g. the sensitivity of the program was set to 7 and the minimum call duration was set to 0.5 ms. We only included bats assigned to the ‘Myosp’ group and assumed these were all Daubenton’s bats since no other *Myotis* species were passing the culverts.

*Manual counting of passing bats*

| **Date** | **east in** | **west in** | **total in** | **east out** | **west out** | **total out** | **total passes** | **east D500x** | **west D500x** | **total D500x** | **turned around** | **over road** |
| --- | --- | --- | --- | --- | --- | --- | --- | --- | --- | --- | --- | --- |
| 21/6/215 | 2 | 18 | 20 | 0 | 0 | 0 | 20 | 4 | 19 | 23 | 2 | 1 |
| 5/7/215 | 4 | 13 | 17 | 0 | 0 | 0 | 17 | 1 | 13 | 14 | 8 | 0 |
| 9/7/215 | 1 | 26 | 27 | 1 | 0 | 1 | 28 | 14 | 31 | 45 | 1 | 1 |
| 17/7/215 | 5 | 11 | 16 | 0 | 0 | 0 | 16 | 6 | 15 | 21 | 0 | 0 |
| 25/7/215 | 7 | 34 | 41 | 2 | 1 | 3 | 44 | 8 | 34 | 42 | 1 | 1 |
| **average** | **3.8** | **20.4** | **24.2** | **0.6** | **0.2** | **0.8** | **25.0** | **6.6** | **22.4** | **29.0** | **2.4** | **0.6** |

SI table 1. Number of bats counted with a handheld bat detector and an infrared camera during the evening half (before midnight) of five nights. ‘East in’ and ‘west in’ indicate the number of bats flying into the tunnels from the southern side, where the lights were installed. The number of bats flying back from the north was very low, and the first few bats passing early in the evening always came from the south. In some occasions, bats turned around or flew over the road. East and west D500x columns contain the number of files with bat calls recorded by the D500x detectors fitted in the culverts during the same evening. During these five evenings, we never observed any other species than *Myotis daubentonii* passing through the culverts.
